# Validity of a self-administered food frequency questionnaire (FFQ) and its generalizability to the estimation of dietary folate intake in Japan

**DOI:** 10.1186/1475-2891-4-26

**Published:** 2005-10-05

**Authors:** Junko Ishihara, Seiichiro Yamamoto, Hiroyasu Iso, Manami Inoue, Shoichiro Tsugane, the JPHC FFQ Validation Study Group

**Affiliations:** 1 Epidemiology and Prevention Division, Research Center for Cancer Prevention and Screening, National Cancer Center, 5-1-1 Tsukiji, Chuo-ku, Tokyo, 104-0051 Japan; 2 Department of Public Health Medicine, Majors of Medical Sciences, Graduated School of Comprehensive Medical Sciences, University of Tsukuba, Ibaraki, Japan; 3 Statistics and Cancer Control Division, Research Center for Cancer Prevention and Screening, National Cancer Center, Tokyo, Japan; 4 Public Health, Department of Social and Environmental Medicine, Graduate School of Medicine, Osaka University, Osaka, Japan

**Keywords:** folate, FFQ, internal validity, external validity, inter-individual variation

## Abstract

**Background:**

In an epidemiological study, it is essential to test the validity of the food frequency questionnaire (FFQ) for its ability to estimate dietary intake. The objectives of our study were to 1) validate a FFQ for estimating folate intake, and to identify the foods that contribute to inter-individual variation of folate intake in the Japanese population.

**Methods:**

Validity of the FFQ was evaluated using 28-day weighed dietary records (DRs) as gold standard in the two groups independently. In the group for which the FFQ was developed, validity was evaluated by Spearman's correlation coefficients (CCs), and linear regression analysis was used to identify foods with large inter-individual variation. The cumulative mean intake of these foods was compared with total intake estimated by the DR. The external validity of the FFQ and intake from foods on the same list were evaluated in the other group to verify generalizability. Subjects were a subsample from the Japan Public Health Center-based prospective Study who volunteered to participate in the FFQ validation study.

**Results:**

CCs for the internal validity of the FFQ were 0.49 for men and 0.29 and women, while CCs for external validity were 0.33 for men and 0.42 for women. CCs for cumulative folate intake from 33 foods selected by regression analysis were also applicable to an external population.

**Conclusion:**

Our FFQ was valid for and generalizable to the estimation of folate intake. Foods identified as predictors of inter-individual variation in folate intake were also generalizable in Japanese populations. The FFQ with 138 foods was valid for the estimation of folate intake, while that with 33 foods might be useful for estimating inter-individual variation and ranking of individual folate intake.

## Introduction

Owing to their ease of administration and low burden on the subject, the assessment of dietary intake in epidemiological studies is often assessed by means of food frequency questionnaires (FFQs) [1]. The chief limitation of FFQs, however, is that serious errors can occur if foods governing inter-individual differences in intake of certain nutrients are omitted from the list. Folate is particularly prone to such error, because it is derived from a variety of foods of both animal and plant origin, not all of which can be included in an FFQ. Foods that contribute to inter-individual differences may fail to be included in food lists, potentially confounding estimation of folate intake by the FFQ.

Another implication of inter-individual variation in the intake of folate or any other nutrient is that such variation may be the underlying determinant of associations between food intake and disease. The preventive effect of certain foods on specific diseases is usually the effect of a particular nutrient contained in the food. Such associations are more likely to be detected when inter-individual differences in intake of the nutrient are larger. Conversely, even if a food contains high levels of a particular nutrient, association will be weak if consumption among individuals is similar. The identification of foods that contribute to inter-individual variation in nutrient intake among the population is therefore an important component of any investigation of nutrients responsible for associations between food intake and disease.

According to the National Nutrition Examination Survey of the United States Population [2] and the National Nutrition Survey in Japan [3], folate intake between the countries is similar among both middle-aged and older age groups. Intake among younger groups, however, is much lower in Japan. This trend is presumably due to differences in the foods that contribute to folate intake. Specifically, folate-fortified food and supplements are large contributors to folate intake for individuals in some Western countries, and questionnaires especially designed to assess folate intake include these items [4,5]. Because folate-fortified foods are not available in Japan and supplement consumption is low, however, folate intake is almost exclusively from natural sources. Nevertheless, little is known about those foods that are the main sources of folate intake among Japanese.

A FFQ was developed and validated for the estimation of dietary intake for the JPHC study. Spearman's correlation coefficients (CCs) between serum folate and folate intake estimated by this FFQ using a biomarker as reference was 0.26 in men [6]. In the present report, we evaluated the validity of this FFQ in the population subgroup for which the FFQ was originally developed using dietary records (DRs) as standard. We then attempted to identify foods that most contributed to folate intake, and those responsible for the differences in intake between individuals. We subsequently repeated these analyses in a second subgroup that was independent of the population for which the FFQ was originally developed, i.e., an external population, to assess its generalizability in Japan. The objectives of this study were: 1) to validate this self-administered FFQ as a means of estimating folate intake, and identify foods that contribute to individual intake and inter-individual variation in folate intake in the population for which the FFQ was developed; and 2) to determine the validity of this FFQ, designed for a study cohort, in estimating the intake of folate and identifying foods that predict inter-individual variation in folate in the general Japanese population.

## Materials and methods

### Study Subjects

**Figure 1:**
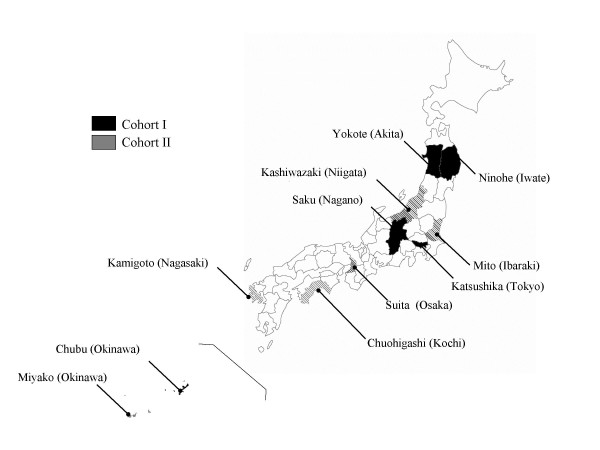
Study sites of the JPHC Study.

Two validation studies of the FFQ were conducted in a subsample of participants in the JPHC Study, a large population-based prospective study that involved medical examination of the study participants. The target population of the JPHC Study consisted of two cohorts, the first started in 1990 (Cohort I) and the second in 1993 (Cohort II). The aim of the cohort study was to investigate the association between various lifestyle factors such as diet and chronic diseases. Despite Japan's small geographical area, considerable regional variation in diet is seen, and study sites were selected to be representative of the whole country. Cohort I was drawn mainly from the northeast part of the country and Cohort II from the southwest (Figure 1). The study design and participants in the entire cohort have been described previously [7].

The present FFQ validation study was conducted in subsamples of Cohort I and Cohort II. The Cohort I study was initiated in February 1995 to validate the FFQ for use in a 5-year follow-up survey [8], given that it was originally developed based on data from 3-day weighed DRs in a random sample from this Cohort [9], while the Cohort II study was to evaluate the generalizability of the FFQ independent of the population for which it was originally developed [10]. Respective numbers and recruitment areas were 247 volunteers from the Ninohe, Yokote, Saku and Chubu (previously named Ishikawa) public health center areas, and 392 volunteers from Mito, Kashiwazaki, Chuo-higashi, Kamigoto, Miyako and Suita. For the present report, we analyzed the data of the 215 and 350 subjects in Cohorts I and II, respectively, for whom the 28-day DR and FFQ data were complete.

### Data Collection

**Figure 2:**
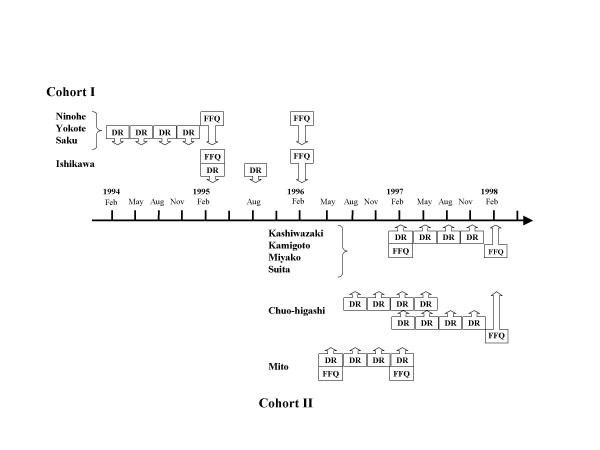
Sequence of data collection for the JPHC FFQ Validation Study.

Data collection has been described in detail elsewhere [8,10]. In brief, each subject completed two FFQs and 28-day DRs (Figure 2). The first FFQ was administered to provide data to compare with the second FFQ as a means of evaluating reproducibility, and the second FFQ was administered to obtain data to compare with the DRs to evaluate its validity. Only data from the second FFQ for validity has been used in this paper.

DRs were collected over 7 consecutive days in each of the 4 seasons, except in Chubu (2 seasons). Local dietitians instructed the subjects to weigh all foods and beverages with the scales and measuring utensils provided, and to record results in a specially designed booklet. The subjects in Cohort I, however, were instructed to use standardized portion sizes for some foods that were difficult to weigh (semi-weighed DRs). The subjects described each food, method of preparation, and the names of the dishes in detail. They also reported all dietary supplements used, if any. At the end of each season, the DRs were reviewed in a standardized manner, and each food was coded by local dietitians.

The self-administered semi-quantitative FFQ consisted of 138 food items and 14 supplementary questions on dietary habits and use of supplements. The validity of the questionnaire in regard to the intake of energy, other nutrients and foods, as well as the use of dietary supplements is described elsewhere [10-13].

Dietary intakes of folate according to the DRs and the FFQ were calculated using the Standardized Tables of Food Composition, 5th ed. [14]. Mean daily intake of folate for the 28 days (14 days in Chubu) was calculated based on the DRs of each subject. Because none of the subjects used folic acid supplementation, dietary supplements were not included in the calculation.

### Statistical analysis

Means and standard deviation of folate intakes from the DRs and FFQ were calculated by sex for Cohort I and Cohort II. Spearman's rank CCs were calculated for crude intake and energy-adjusted intake by the residual method, and were corrected for the attenuating effect of random intra-individual error (deattenuation) in Cohort I subjects to evaluate internal validity in the population for which the FFQ was developed. Deattenuation was done using the following formula: Deattenuated , where r is the observed correlation, λ_x _is the ratio of intra- to inter-subject variation, and n_x _is number of dietary records for each subject [15]. The same analysis was performed for Cohort II subjects to evaluate external validity

The percentage contribution of each food to total folate intake was computed based on the DRs of Cohort I subjects, and the 20 foods contributing most were listed based on their percentage contribution. Percentage contributions of the same 20 foods in the DRs of Cohort II were calculated and their actual rank of percentage contribution in Cohort II was determined.

Linear regression analysis with stepwise selection was used to identify foods that contributed to inter-individual variation, with folate intake from each food item according to the FFQ of Cohort I used as the explanatory variable, and total folate intake according to the DRs as the response variable. A model (partial) R-square value for the selected food items was computed. Cumulative mean intake from each food item on the list was calculated, and compared to total intake according to the DRs by Spearman's CCs to evaluate validity. Cumulative intake from the same food items in the Cohort II subjects and their CCs from the DR data were calculated to evaluate external validity.

## Results

Daily folate intake as assessed by DRs and FFQ as well as the Spearman's rank CCs between the two measurements by cohort group and sex are shown in Table 1. Mean daily intake of folate based on the FFQ in Cohort I was significantly overestimated compared to the DR data. Both crude and adjusted CCs were higher in men than women in Cohort I but were similar in Cohort II (Table 1).

**Table 1:** Folate intake (μg/day) assessed by the dietary records and food frequency questionnaire, and their correlation coefficients.

	DR^1^			FFQ^2^				Spearman correlation		
				
	Mean ± SD	Median	Range	Mean ± SD	Median	Range	% difference of mean	Crude	Energy-adjusted^3^	Deattenuated
Cohort I										
Men (n = 102)	425 ± 103	427	210–735	473 ± 231	444	119–1807	11	0.49	0.40	0.57
Women (n = 113)	389 ± 106	380	153–667	476 ± 287	419	146–1978	22	0.29	0.35	0.47
										
Cohort II										
Men (n = 174)	467 ± 156	443	197–1280	421 ± 190	370	85–1178	-10	0.33	0.50	0.63
Women (n = 176)	426 ± 112	417	198–980	454 ± 237	397	4–1498	7	0.42	0.48	0.63

^1 ^DR, dietary records

^2 ^FFQ, food frequency questionnaire.

^3 ^Folate was adjusted for total energy intake using the residual method.

The 20 foods that made the greatest contribution to total folate according to the DR in Cohort I are listed in Table 2. The list consists of various foods, mainly from plant sources such as vegetables, with spinach making the highest contribution followed by rice, green tea, cabbage, eggs and beer. These 20 foods contributed 55.2% of total intake in men and 52.9% in women. The contribution of the same 20 foods to folate intake in Cohort II subjects was 44.9% for men and 43.2% for women. *Gyokuro*, a type of green tea, made the second highest contribution in Cohort II, but was not among the 20 in Cohort I. Other food items with the highest contribution in Cohort II but not Cohort I were *kamairi-cha *(pan-fried green tea), bread, tomatoes, and pumpkins in both sexes; purple laver in men; and sweet potatoes and *komatsuna *(a green leafy vegetable) in women. When both kinds of green tea were excluded, however, these foods accounted for only 4.6% of total intake in men and 6.2% in women.

**Table 2:** Foods that contribute to folate intake and their cumulative percentage contribution to total intake as assessed by dietary records.

Men	Cohort I		Cohort II		Women	Cohort I		Cohort II	
			
Food item	Rank^1^	%	Rank^2^	%	Food items	Rank^1^	%	Rank^2^	%
Spinach, leaves	1	6.5	3	5.4	Spinach, leaves	1	7.1	3	5.6
Well-milled rice	2	6.4	4	5.0	Green tea, *sencha*, infusion	2	6.1	1	10.1
Green tea, *sencha*, infusion	3	5.6	1	9.1	Cabbage, head	3	4.7	5	3.4
Cabbage, head	4	4.9	5	3.6	Well-milled rice	4	4.5	4	3.6
Chicken eggs, whole	5	3.8	6	3.6	Chicken eggs, whole	5	3.5	6	3.1
Beer	6	3.3	7	3.0	Aspargus, shoots	6	2.8	36	0.7
Radish, root with skin	7	2.6	8	2.5	Chiken offal, liver	7	2.5	15	1.2
Pork offal, liver	8	2.4	35	0.7	Radish, root with skin	8	2.5	7	2.4
Aspargus, shoots	9	2.3	34	0.7	Bracken, young shoots	9	1.9	72	0.3
*Natto, itohiki-natto *(whole fermented using Bacillus natto)	10	2.0	13	1.4	*Natto, Itohiki-natto *(Whole fermented using Bacillus natto)	10	1.9	10	1.7
*Miso*, rice- *koji miso*, dark yellow type	11	1.9	46	0.5	Broccoli, florets	11	1.8	8	2.0
Chiken offal, liver	12	1.8	12	1.4	*Miso*, rice- *koji miso*, dark yellow type	12	1.8	55	0.4
Chinese cabbage, head	13	1.8	11	1.7	Chinese cabbage, head	13	1.7	11	1.6
Bracken, young shoots	14	1.6	67	0.3	Ordinary liquid milk	14	1.5	20	1.1
Broccoli, florets	15	1.6	9	1.8	*Shoyu*: soy sauce, *Koikuchi-shoyu *(Common type)	15	1.5	23	1.0
*Shoyu*: soy sauce, *Koikuchi-shoyu *(Common type)	16	1.6	17	1.1	Head letucce, crisp type, head	16	1.5	22	1.1
Carrots, European type, root with skin	17	1.4	16	1.1	Purple laver, toasted	17	1.5	18	1.1
Potatoes, tuber	18	1.3	23	0.9	Pork offal, liver	18	1.4	34	0.7
Head lettuce, crisp type, head	19	1.3	22	1.0	Carrots, European type, root with skin	19	1.4	17	1.1
Leaf mustard, leaves	20	1.2	75	0.2	Potatoes, tuber	20	1.3	26	0.9
									
Total		55.2		44.9			52.9		43.1

^1 ^Food items are listed in order of % contribution to total folate intake based on dietary records of Cohort I (by rank in Cohort I).

^2 ^Rank of % contribution to total folate intake determined on the basis of the dietary records of Cohort II.

The foods that best predicted inter-individual variation in dietary folate and the validity (correlation coefficients) of folate intake based on those foods are shown in Table 3. A total of 33 foods are listed with the cumulative R-square value of 0.59. The food that best predicted variation of intake was green tea, which contributed 12–15% of total intake. No other food predictive of variation contributed more than 1% of total folate intake. The cumulative folate intake from the 33 foods contributed approximately 30% of total intake in both men and women. The CC of intake from the 33 foods in the internal population (Cohort I) was 0.46 in men and 0.28 in women (indicated as "internal" in Table 3), and had approximately the same level of validity as the data from the full 138-food FFQ.

**Table 3:** Foods most predictive of inter-individual variation in dietary folate, and their correlation coefficients with intake from DR.

Foods selected by regression analysis^1^	Male and female		Male						Female					
	
	Partial	Cumulative	Cohort I (internal)			Cohort II (external)			Cohort I (internal)			Cohort II (external)		
			
	R-Square^1^	R-Square^1^	Cumulative		Spearman^3^	Cumulative		Spearman^3^	Cumulative		Spearman^3^	Cumulative		Spearman^3^
			mean intake		correlation	mean intake		correlation	mean intake		correlation	mean intake		correlation
			μg/day	(%)^2^		μg/day	(%)^2^		μg/day	(%)^2^		μg/day	(%)^2^	
Green tea (*sencha*)	0.096	0.096	72	(15.3)	0.31	55	(13.0)	0.28	55	(11.6)	0.17	66	(14.6)	0.25
Dried small fish	0.070	0.166	73	(15.4)	0.33	55	(13.1)	0.27	56	(11.7)	0.19	67	(14.7)	0.26
Horse mackerel, sardine	0.039	0.204	74	(15.7)	0.37	57	(13.5)	0.27	57	(12.0)	0.20	68	(15.0)	0.24
Cake	0.031	0.236	74	(15.7)	0.37	57	(13.6)	0.27	58	(12.1)	0.19	69	(15.1)	0.24
*Miso *soup	0.030	0.266	84	(17.8)	0.37	62	(14.8)	0.28	66	(13.8)	0.24	73	(16.1)	0.27
Luncheon Meat	0.032	0.298	84	(17.8)	0.38	62	(14.8)	0.28	66	(13.8)	0.24	73	(16.1)	0.27
Ham, loin	0.022	0.320	84	(17.8)	0.38	62	(14.8)	0.29	66	(13.8)	0.24	73	(16.1)	0.27
Cream for coffee	0.014	0.335	85	(17.9)	0.37	63	(14.9)	0.28	66	(13.9)	0.24	74	(16.2)	0.27
Stewed pork, Western style	0.012	0.346	85	(17.9)	0.37	63	(15.0)	0.28	66	(14.0)	0.24	74	(16.2)	0.27
Mayonnaise	0.011	0.357	85	(17.9)	0.37	63	(15.0)	0.28	66	(14.0)	0.24	74	(16.2)	0.27
Worcester sauce	0.013	0.370	85	(17.9)	0.37	63	(15.0)	0.28	66	(14.0)	0.24	74	(16.2)	0.27
*Kamaboko *(fish paste product)	0.014	0.384	85	(18.0)	0.37	63	(15.0)	0.28	67	(14.0)	0.24	74	(16.3)	0.27
Lettuce	0.011	0.395	86	(18.3)	0.37	64	(15.3)	0.30	68	(14.3)	0.25	75	(16.5)	0.27
Bean sprouts	0.015	0.410	88	(18.7)	0.37	66	(15.7)	0.29	71	(14.9)	0.23	77	(16.9)	0.26
Peaches	0.017	0.427	89	(18.7)	0.38	66	(15.8)	0.29	71	(14.9)	0.23	77	(17.0)	0.26
Sausage, Wieners	0.011	0.437	89	(18.7)	0.38	66	(15.8)	0.29	71	(14.9)	0.23	77	(17.0)	0.26
Chocolate	0.009	0.446	89	(18.8)	0.38	67	(15.9)	0.29	71	(15.0)	0.23	78	(17.1)	0.26
Octopus	0.008	0.455	89	(18.8)	0.38	67	(15.9)	0.29	71	(15.0)	0.23	78	(17.1)	0.26
Salted fish	0.008	0.463	90	(19.1)	0.38	68	(16.0)	0.30	73	(15.2)	0.23	78	(17.2)	0.26
Apples	0.010	0.473	92	(19.5)	0.38	69	(16.3)	0.31	75	(15.8)	0.24	80	(17.6)	0.26
Sweet pepper	0.012	0.485	94	(19.8)	0.39	70	(16.6)	0.31	77	(16.1)	0.26	81	(17.9)	0.27
*Udon*	0.011	0.496	95	(20.0)	0.39	71	(17.0)	0.30	78	(16.3)	0.25	82	(18.1)	0.27
Grilled chicken	0.014	0.510	95	(20.1)	0.39	72	(17.1)	0.30	78	(16.4)	0.25	83	(18.2)	0.27
Pickled plums	0.009	0.519	95	(20.1)	0.39	72	(17.1)	0.30	78	(16.4)	0.25	83	(18.2)	0.27
Papaya	0.009	0.528	96	(20.2)	0.39	73	(17.3)	0.29	79	(16.5)	0.25	83	(18.4)	0.27
Black tea	0.007	0.536	96	(20.4)	0.39	73	(17.4)	0.30	79	(16.7)	0.24	84	(18.5)	0.28
Green tea (*bancha, genmaicha*)	0.007	0.542	105	(22.1)	0.42	83	(19.8)	0.33	88	(18.6)	0.25	95	(21.0)	0.35
Chicken liver	0.007	0.550	122	(25.8)	0.45	98	(23.2)	0.32	101	(21.2)	0.28	107	(23.6)	0.34
Bananas	0.008	0.557	126	(26.6)	0.46	101	(24.1)	0.32	104	(21.9)	0.27	111	(24.3)	0.34
Rice mixed with other grains	0.005	0.562	128	(27.1)	0.45	103	(24.5)	0.33	107	(22.5)	0.27	112	(24.8)	0.34
Well-milled rice	0.011	0.573	152	(32.2)	0.49	124	(29.5)	0.30	125	(26.2)	0.29	128	(28.3)	0.34
*Yushi-dofu*	0.006	0.579	153	(32.3)	0.49	125	(29.6)	0.30	125	(26.3)	0.29	129	(28.4)	0.34
Bitter gourds	0.011	0.590	158	(33.3)	0.46	128	(30.5)	0.30	130	(27.2)	0.28	132	(29.1)	0.35

^1 ^Foods were selected by stepwise regression analysis using data from the food frequency questionnaire of Cohort I men and women. Partial and cumulative R-Square values were calculated in the process of performing the regression analysis.

^2 ^Percent of total folate according to the food frequency questionnaire.

^3 ^Spearman's correlation coefficients between cumulative intake and total intake based on dietary records

When the same 33-food list was used to compute intake in the external population (Cohort II), the cumulative folate intake contribution was again 30% (indicated as "external" in Table 3), and CC was 0.30 in men and 0.35 in women, showing approximately the same level of validity as in the internal population.

## Discussion

In this study, we evaluated the validity of a FFQ as a means of estimating dietary folate intake in the population for which the FFQ was originally developed. We also attempted to identify foods that differentiated the level of folate intake in individuals by stepwise regression, and tested the validity of assessing folate intake based on the intake of these foods. The results were also cross-validated in an independent population to evaluate generalizability.

Validity of the FFQ in estimating folate intake was moderate in both the internal and external populations. In previous studies, the validity of questionnaires in estimating energy-adjusted dietary folate intake varied from 0.2 to 0.6, depending on the study population [15-21]. In studies that reported intake from the diet and from dietary supplements separately, CC was 6–21% higher for folate intake that included supplements than for intake from diet alone. Although none of the subjects used supplements that contained folic acid, which had only just become available at the time of the study, the validity of our FFQ in estimating folate from the diet was relatively high, probably because the dietary folate intake of our subjects was as high as that of supplement users in some of the previously cited studies.

Although the largest proportion of folate intake was from vegetables, these did not necessarily explain the differences in intake between individuals. For example, spinach, which is very rich in folate and was one of the highest contributors to mean folate intake, could not explain inter-individual variation because it was consumed by almost every subject. In contrast, green tea contributed greatly to both individual intake and to inter-individual variation, probably because consumption was strongly dependent on individual preference. Although the analysis provided us with information about foods that predicted inter-individual variation in folate intake, some foods which had a moderate partial R-square value contributed less than 0.1% to total folate intake, such as luncheon meat, ham, and so on. These may have been selected by chance alone. In this kind of analysis, even unimportant contributors to the cumulative R-square value may be statistically significant, but can nevertheless be ignored [1]. In any case, it is noteworthy that individuals could be ranked by folate intake based on only 33 foods, with the same level of validity as with the long FFQ.

Any analysis of the possible effects of folate intake on disease also requires analysis of the effect of food items which contribute to total intake and inter-individual variation of folate. It is of great research interest to determine whether the association between food intake and disease is the result of folate intake. For example, we might hypothesize that folate intake may help explain the protective effect of green tea on gastric cancer described in the recent report of the JPHC study [22]. The mechanism of carcinogenesis through DNA instability and methylation abnormalities as a result of folate deficiency has been studied in animal and in vitro studies [23-25], and an association between folate and gastric cancer has been reported in a number of case-control studies [26-29]. Other prospective studies, however, have failed to show a consistent association between green tea and gastric cancer [30-32]. We speculate that the association was strong in the JPHC population owing to the large contribution of green tea to the variation in folate intake, which was not seen in the other populations.

One of the strengths of our study is the precision of the reference data. The ratio of intra- to inter-individual variation in our data was somewhat higher (1.9–4.8) than in several studies in Western countries [33,34]. When intra-individual variation is larger, an increased number of dietary assessment days is required to obtain a valid standard. Although intra-individual variation among our subjects was high, we had more than a sufficient numbers of days (28 days) of data to represent the true intake of the individuals, because the number of DR days needed to estimate true intake within 20% of the true mean with our data was only about 17 to 19 days according to our analysis. By comparison, the greatest number of days of dietary assessment used as standard in previous studies was 14 days [35].

In addition, our analysis is unique because we used regression analysis to identify the foods most predictive for inter-individual variation in dietary folate, and then evaluated both the internal and external validity of the intake of those foods. It is important to test external validity, because there is no assurance that explanatory variables selected by regression analysis are valid in an external population [1]. To our knowledge, this is the first study to attempt to identify foods that contribute to inter-individual variation of folate intake in Japan, where folate intake is almost exclusively from natural sources. We developed a list of foods that contribute to inter-individual variation in folate intake in the population for which FFQ was originally developed, and tested the generalizability of the results to an external population. The two populations covered various geographic areas throughout Japan. Our results imply the possibility that a shorter questionnaire which specifically targets folate intake in Japanese populations can be developed.

One limitation of our study is that because the subjects needed to be highly motivated to complete the 28-day DRs, they were not a randomly selected sample. Mean folate intake based on DRs was slightly higher than in the entire cohort, probably because the validation study subjects were likely more health conscious and consumed more vegetables. If all subjects had consumed a similar amount of certain foods, inter-individual variation in the food might have been falsely low. The generalizability of questionnaire results needs to be established with care.

## Conclusion

Our FFQ is valid for estimating folate intake and is generalizable to the Japanese population. Although some foods such as spinach, rice and cabbage contributed more to total intake of folate, they did not necessarily contribute to inter-individual variation. Validity of the estimation of folate intake based on intake of the 33 foods that most contributed to inter-individual variation was about the same as that based on the original 138-food FFQ. Although folate is contained in a wide variety of foods, 33 foods in the FFQ were sufficient to account for the inter-individual variation of intake and ranking of individuals' intake in Japan. We concluded that the FFQ with 138 foods was valid for the estimation of folate intake, and that the FFQ with 33 foods might be useful for estimating inter-individual variation and the ranking of individual folate intake.

## Competing interests

The author(s) declare that they have no competing interests.

## List of Abbreviations

FFQ: food frequency questionnaire

DR: dietary record

CC: correlation coefficients

JPHC Study: Japan Public Health Center-based prospective Study

## Authors' contributions

JI performed the data analysis and drafted the manuscript

SY participated in the design of the study, coordinated the study and helped with analysis and with the preparation of the manuscript

HI helped with analysis and with the preparation of the manuscript

MI helped with analysis and with the preparation of the manuscript

ST participated in the design of the study and helped to draft the manuscript. He was principal investigator of the JPHC Study

All authors have read and approved the final manuscript.
